# Probabilistic mapping of lymph node metastasis in epithelial ovarian cancer: a retrospective cohort study using Bayesian network analysis

**DOI:** 10.3389/fonc.2026.1817368

**Published:** 2026-05-18

**Authors:** Huishan Xu, Chuling Wu, Yuying Chen, Linxiang Wu, Qin Ling, Siqi Yang, Huiling Lai, Weijia Wen, Hao Tan, Shuzhong Yao, Tingting Sun, Guofen Yang

**Affiliations:** 1 Department of Gynecology, the First Affiliated Hospital of Sun Yat-sen University, Guangzhou, China; 2 Department of Obstetrics and Gynaecology, Sun Yat-sen Memorial Hospital of Sun Yat-Sen University, Guangzhou, China; 3 Department of Gynecology, The Sixth Affiliated Hospital, Sun Yat-Sen University, Guangzhou, China

**Keywords:** Bayesian network diagram, clinicopathological factors, epithelial ovarian cancer, lymph node metastasis, regional distribution map

## Abstract

**Objective:**

To characterize the anatomical distribution, potential metastatic pathways, and clinical factors associated with lymph node metastasis (LNM) in epithelial ovarian cancer (EOC), providing evidence to guide individualized surgical management.

**Methods:**

A retrospective analysis was performed on 654 EOC patients who underwent radical surgery. Metastatic lymph nodes were categorized into 14 anatomical regions, with LNM rates calculated and distribution maps generated. Bootstrap-derived Bayesian methods were used to construct a metastatic pathway model. Univariate and multivariate regression analyses were conducted to identify risk factors for LNM, and Bayesian conditional probability was applied to analyze the association between these factors and LNM extent.

**Results:**

LNM was identified in 291 of 654 patients (44.50%), with the highest involvement in the left para-aortic region (54.3%) and the lowest in the right superficial inguinal region (10.9%). The Bayesian model revealed three core metastatic pathways: 1) internal/external iliac lymph nodes → common iliac lymph nodes; 2) primary tumor → para-aortic lymph nodes; 3) obturator lymph nodes → inguinal/presacral lymph nodes. Incorporating clinical variables into the Bayesian framework highlighted key determinants of metastatic extent: tumor size, histological grade, and patient age. This integrated analysis quantified node-specific metastasis probabilities and identified high-risk lymphatic routes, providing a patient-specific roadmap for targeted lymphadenectomy and precision surgical planning.

**Conclusion:**

This study provides a probabilistic characterization of lymph node involvement patterns in EOC based on retrospective observational data. The findings may support hypothesis generation for risk-adapted nodal assessment and warrant further validation in prospective studies.

## 1 Introduction

Ovarian cancer (OC) ranks among the most lethal gynecological malignancies worldwide, with a dismal 5-year overall survival (OS) rate of approximately 46% due to its high propensity for late-stage diagnosis and aggressive metastatic behavior (1, 2). Epithelial ovarian cancer (EOC) constitutes the predominant histological subtype, accounting for over 90% of all OC cases, and its clinical management remains a major challenge in gynecologic oncology (3). Lymph node metastasis (LNM) is a key determinant of disease progression and staging according to the International Federation of Gynecology and Obstetrics (FIGO) classification system and plays an important role in therapeutic decision-making (4). Lymph node involvement may be identified in a subset of patients initially presumed to have early-stage disease following surgical staging, while its incidence is substantially higher in advanced-stage disease, underscoring the clinical relevance of nodal assessment across different disease settings. It should be noted, however, that FIGO stage I disease is by definition node-negative; thus, reported rates of nodal involvement in “early-stage” cohorts generally reflect cases that were clinically staged preoperatively but subsequently upstaged after pathological evaluation (5–7).

The primary objective of cytoreductive surgery in EOC is the complete gross resection of visible disease (8). Although systematic pelvic and para-aortic lymphadenectomy has historically been incorporated into surgical management in selected settings, its role has become increasingly debated, particularly in advanced-stage disease. Current clinical practice generally favors a more selective approach to lymph node dissection based on intraoperative findings and the presence of clinically suspicious or bulky nodes. This evolving perspective is supported by accumulating evidence questioning the survival benefit of systematic lymphadenectomy in patients without apparent nodal involvement. For instance, a landmark study by Ignace Vergote et al. demonstrated that while systematic lymphadenectomy improved progression-free survival (PFS), it did not confer an overall survival (OS) benefit in advanced EOC (9). More notably, the LION trial further confirmed that systematic lymphadenectomy conferred no meaningful improvements in either PFS or OS among lymph node-negative advanced EOC patients, while significantly increasing the risk of postoperative complications such as lower limb lymphedema, pelvic lymphocyst, and vascular injury (10). These complications can lead to chronic morbidity and impaired quality of life (9, 11–13). Taken together, these findings highlight the importance of developing more refined, risk-adapted strategies for nodal assessment rather than relying on routine extensive lymph node dissection.

Despite decades of research, critical gaps persist in our knowledge of EOC LNM. Noninvasive diagnostic modalities, including serum biomarkers such as CA125 and cross-sectional imaging techniques (computed tomography and magnetic resonance imaging), lack sufficient sensitivity and specificity to reliably detect occult nodal disease, potentially leading to both over-treatment and under-treatment (7, 14–19). Sentinel lymph node (SLN) mapping, first introduced for EOC in 1991 (20), offers a potential minimally invasive alternative to systematic lymphadenectomy; however, technical limitations (e.g., variable SLN detection rates, inconsistent tracer selection) and concerns about false-negative results have hindered its widespread clinical adoption (20–22). Moreover, most existing studies investigating LNM in EOC have relied on descriptive analyses or conventional regression-based methods, which primarily evaluate associations between individual clinical variables and nodal status (15, 18, 23). Such approaches are limited in their ability to capture the complex interrelationships and co-occurrence patterns among different lymph node regions and therefore may not adequately reflect the heterogeneous and non-random nature of lymphatic dissemination.

To address these limitations, we applied a Bayesian network framework, a probabilistic graphical modeling approach that enables the characterization of conditional dependencies among variables within complex systems using observational data. In this retrospective study, we aimed to characterize the regional distribution of lymph node involvement and to model probabilistic patterns of nodal co-occurrence, rather than to infer definitive metastatic pathways or directly inform surgical decision-making. Specifically, we sought to (1) systematically describe the anatomical distribution of lymph node metastasis in EOC, (2) identify patterns of conditional dependency among nodal regions, and (3) estimate region-specific probabilities of nodal involvement using Bayesian network modeling. By integrating anatomical mapping with probabilistic analysis, this study provides a hypothesis-generating framework for understanding lymphatic dissemination in EOC and may contribute to improved risk stratification and the design of future prospective studies.

## 2 Patients and methods

### 2.1 Study population

Patients diagnosed with epithelial ovarian cancer at the First Affiliated Hospital of Sun Yat-sen University between January 2014 and December 2022 were included in this retrospective study. The inclusion criteria were as follows: 1) initial diagnosis of epithelial ovarian cancer and performance of surgery treatment at the First Affiliated Hospital of Sun Yat-sen University; 2) confirmation of epithelial ovarian cancer by postoperative pathology; 3) available data on lymph node status; and 4) accessible clinical information, including results of preoperative ultrasound and blood biochemistry tests. The exclusion criteria were as follows: 1) initial diagnosis or surgical treatment received at other hospitals; 2) lack of imaging results (CT/PET-CT/MRI) or postoperative pathology results for lymph node status; 3) concurrent diagnosis of other malignant tumors; and 4) incomplete medical records. A total of 654 patients who met these criteria were included in the study (**Supplementary Figure 1**). The full cohort of 654 patients was used to analyze factors associated with the presence of lymph node metastasis. For the analysis of lymph node distribution patterns and Bayesian network modeling, a subset of patients who underwent systematic pelvic and para-aortic lymphadenectomy with pathologically confirmed lymph node metastasis was further included. In our institution, patients with presumed early-stage disease (FIGO I–II) underwent standard surgical staging procedures, including systematic pelvic and para-aortic lymphadenectomy, whereas patients with advanced-stage disease (FIGO III–IV) underwent cytoreductive surgery with lymph node assessment performed either systematically or selectively based on intraoperative findings. To ensure the reliability of nodal distribution analysis, only patients who received systematic lymphadenectomy were included in the mapping and modeling analyses. This study was approved by the Institutional Review Board (IRB) of the First Affiliated Hospital of Sun Yat-sen University, and the requirement for written informed consent was waived due to the retrospective nature of the study and the use of anonymized data.

### 2.2 Clinicopathological data collection and processing

Clinicopathological data were systematically collected to identify potential predictors of LNM, including age at diagnosis, menstrual status (premenopausal/postmenopausal), parity (number of completed pregnancies), personal history of malignancy, history of benign gynecological diseases, family history of cancer, preoperative biomarkers (serum concentrations of CA125 and HE4), tumor size (maximal tumor diameter on preoperative imaging), histological grade, pathological subtype, FIGO stage ascites volume (quantified on preoperative imaging), tumor lateralization, and administration of neoadjuvant chemotherapy (NACT).Patients were stratified into two cohorts: lymph node metastasis-positive (LN+) group and metastasis-negative (LN–) group, with status confirmed by postoperative histopathology. To optimize clinical utility and address nonlinear associations, continuous variables (CA125, HE4, ascites volume) were dichotomized using receiver operating characteristic (ROC) curve analysis. The optimal cut-off value for each variable was determined by maximizing the Youden index (24, 25). These cut-off values were derived from the same dataset used for subsequent analyses and may therefore be subject to potential overfitting. Variables with P < 0.05 in univariate analysis were entered into the multivariable logistic regression model, and a forward stepwise selection procedure was applied to identify independent factors. Given the retrospective design, this approach was considered exploratory. Missing data (accounting for < 5% of all cases) were handled via complete-case analysis.

### 2.3 Anatomical division of lymph nodes

**Figure 1:**
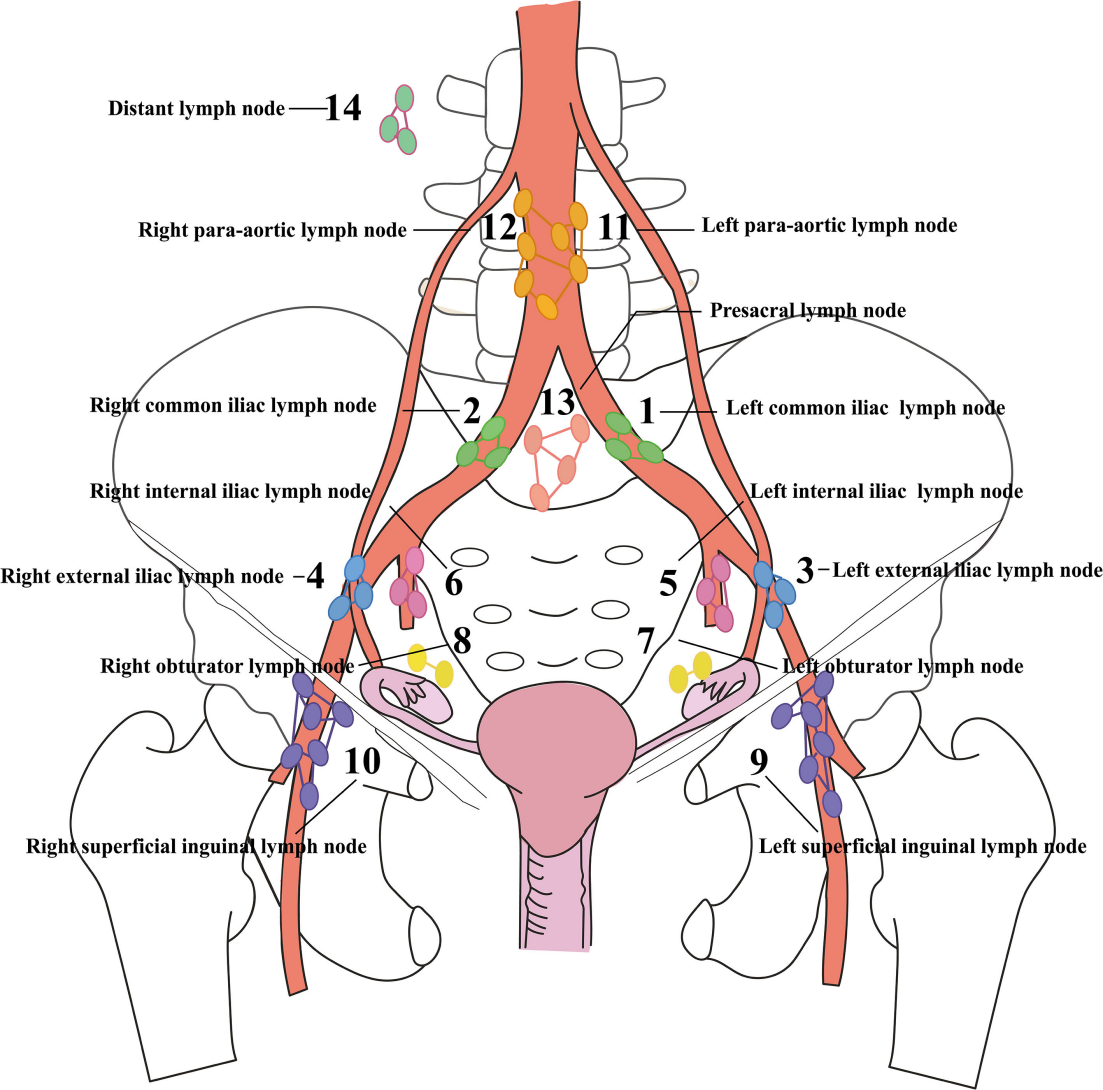
Anatomical map of positive lymph node (LN) regions. Fourteen lymph node regions are numbered in a predefined anatomical order. Each color represents a distinct LN zone, and the anatomical name corresponding to each region is labeled adjacent to its serial number.

In this study, lymph nodes were classified into 14 anatomical regions in accordance with the 2014 FIGO staging system and the ESGO ovarian cancer surgery guideline (4, 26) (**Figure 1**): 1) left common iliac, 2) right common iliac, 3) left external iliac, 4) right external iliac, 5) left internal iliac, 6) right internal iliac, 7) left obturator, 8) right obturator, 9) left superficial inguinal, 10) right superficial inguinal, 11) left para-aortic, 12) right para-aortic, 13) presacral, and 14) distant lymph nodes (e.g., mesenteric or supraclavicular lymph nodes). Pelvic lymph nodes (Regions 1-8,13) were defined as follows: laterally bounded by the external iliac vessels, medially by the umbilical artery, inferiorly by the obturator nerve, superiorly by the bifurcation of the common iliac artery, and anteriorly by the posterior wall of the wall of the bladder. Para-aortic lymph nodes included Regions 11 and 12, with the superior boundary at the bifurcation of the renal artery and the inferior boundary at the bifurcation of the common iliac artery (27). Distant lymph nodes encompassed all other lymph node regions, such as supraclavicular, mediastinal, or inguinal lymph nodes.

Lymph node metastasis (LNM) was defined based on postoperative histopathological evaluation by two gynecologic pathologists via examination of paraffin-embedded sections of resected lymph nodes, metastasis was defined as the presence of tumor cell in nodal tissue. Imaging findings (including CT, MRI, or PET-CT) were used only for preoperative assessment and patient selection, but were not used to define lymph node metastasis in the primary analyses (28).

### 2.4 Bayesian networks and co-occurrence probabilities

We constructed a Bayesian network (BN) model to characterize the conditional dependencies of LNM across different anatomical regions. A Bayesian network is a probabilistic graphical model represented as a directed acyclic graph (DAG), which expresses causal or conditional relationships among variables. It is particularly suitable for modeling complex interactions between nodes when predefined biological pathways are unclear (29). In this study, each node in the graph represents a specific lymph node region (Regions 1-14), and each directed edge indicates a conditional dependency from one region to another. The network structure was learned from the data using a Hill-Climbing algorithm, with the Bayesian Information Criterion (BIC) employed as the scoring function (30). Maximum Likelihood Estimation (MLE) was then used to compute the Conditional Probability Table (CPT) for each node, and probabilistic inference was performed to calculate the posterior probability of metastasis in one region given the presence of metastasis in other regions. The posterior probability of each edge—defined as the proportion of bootstrap iterations in which the edge appeared—was used to estimate the confidence in the conditional dependency between two regions. These bootstrap-based posterior probabilities were subsequently treated as proxies for the transition likelihood of metastasis from one region to another (31).

To identify frequently co-involved lymph node regions, we also calculated the co-occurrence probability between each pair of lymph node regions. This probability is defined as the joint probability that two regions are simultaneously involved in metastasis in the same patient. It was computed as the proportion of patients with metastasis in both regions relative to the total number of patients, thus reflecting a non-causal correlation based on observed data (32).

To enhance the robustness of metastatic pathway prediction, we integrated the posterior probabilities derived from the Bayesian network (BN) with the co-occurrence probability (33, 34). A lymph node region was considered to be part of potential metastatic pathway if it exhibited both a high posterior probability (from the BN) and a high co-occurrence frequency with other metastatic lymph node regions.

## 3 Statistical analysis

Statistical analyses were performed using SPSS software (version 25.0) and R (version 4.2.3). Clinicopathological variables were summarized descriptively. Non-normally distributed continuous variables were reported as medians with interquartile ranges (IQRs), and categorical variables as counts and percentages. For group comparisons, the Chi-square test was used for categorical variables, and the Mann-Whitney U test was used for continuous variables. Univariate and multivariate logistic regression analyses were conducted to identify independent risk factors associated with lymph node metastasis (LNM). To quantify and map lymph node region metastasis, the number of resected lymph node was reported as median (minimum, maximum). Bayesian network modeling was implemented using the bnlearn package in R for structure learning, parameter estimation, and probabilistic inference (30). Co-occurrence probabilities between lymph node regions were calculated based on the observed joint frequencies of metastasis. A P value <0.05 was considered statistically significant in all analyses.

## 4 Results

### 4.1 Clinicopathological characteristics of patients

A total of 654 patients with epithelial ovarian cancer (EOC) were included in the analysis. Among them, lymph node metastasis (LNM) was identified in 291 patients (44.5%), while 363 patients (55.5%) had no evidence of nodal involvement based on postoperative histopathological evaluation. Univariable logistic regression analysis was performed to evaluate associations between clinicopathological variables and LNM status. In total, 13 candidate variables (age at diagnosis, menstrual status, number of completed pregnancies history of benign gynecological diseases, serum concentrations of CA125 and HE4, tumor size, histological grade, pathological subtype, FIGO stage, ascites volume, tumor lateralization, and NACT) met this criterion. Variables with statistical significance in univariable analysis were subsequently included in the multivariable logistic regression model (**Tables 1**, **2**). In multivariable analysis, FIGO stage and tumor lateralization remained statistically associated with LNM. As expected, FIGO stage demonstrated a strong association with LNM, reflecting its intrinsic incorporation of nodal status. Tumor lateralization also showed a significant association with LNM and was therefore considered in subsequent analyses. Preoperative neoadjuvant chemotherapy (NACT) was also associated with LNM status in multivariable analysis. To minimize potential treatment-related bias and to ensure that subsequent analyses reflected intrinsic patterns of lymphatic dissemination, patients who had received NACT were excluded from further analyses.

**Table 1:** Clinical characteristics of patients included in this study.

Variables	Total (n = 654)	LNM negative (n = 363)	LNM positive (n = 291)	*P*
Age				
Mean (Q_1_, Q_3_)	53.00 (46.00, 59.45)	51.00 (45.20, 57.00)	55.00 (48.18, 62.95)	<0.001^a^
<50years	231 (35.32)	150 (41.60)	80 (27.49)	<0.001^b^
≥50years	423 (64.68)	212 (58.40)	211 (72.51)	
Personal cancer history				
No	624 (95.41)	337 (92.84)	280 (96.22)	0.377^b^
Yes	30 (4.59)	19 (5.23)	11 (3.78)	
Family cancer history				
No	527 (80.58)	293 (80.72)	234 (80.41)	0.922^b^
Yes	127 (19.42)	70 (19.28)	57 (19.59)	
Benign gynecological diseases				
No	623 (95.41)	337(92.84)	286 (98.62)	<0.001^b^
Yes	30 (4.59)	26 (7.24)	4 (1.36)	
Menstruation				
No	304 (46.48)	185 (50.96)	119 (40.89)	0.010^b^
Yes	350 (53.52)	176 (49.04)	172 (59.11)	
Number of completed pregnancies				
0	50 (7.72)	33 (9.14)	17 (5.92)	0.007 ^b^
1-3	438 (67.59)	255 (70.64)	183 (63.76)	
>3	160 (24.69)	73 (20.22)	87 (30.31)	
CA125 (U/mL) ≥cut-off ^1^				
No	253 (40.03)	199 (57.51)	54 (18.88)	<0.001 ^b^
Yes	379 (59.97)	147 (42.49)	232 (81.12)	
HE4 (U/mL) ≥cut-off ^2^				
No	222 (37.88)	173 (53.23)	50 (18.77)	<0.001 ^b^
Yes	364 (62.12)	152 (46.77)	212 (81.23)	
Ascites (mL) ≥cut-off ^3^				
No	259 (39.60)	172 (47.38)	87 (29.90)	<0.001^b^
Yes	395 (60.40)	191 (52.62)	204 (70.10)	
Tumor Size (mm)				
M (Q_1_, Q_3_)	91.50 (61.00, 130.00)	102.00 (67.00, 138.00)	80.00 (53.50, 112.00)	<0.001 ^a^
<100	357 (55.35)	170 (47.49)	187 (65.16)	<0.001 ^b^
≥100	288 (44.65)	188 (52.51)	100 (34.84)	
Tumor lateralization				
Unilateral	364 (55.66)	254 (69.97)	110 (37.80)	<0.001 ^b^
Bilateral	290 (44.34)	109 (30.03)	181 (62.20)	
Stage (FIGO)				
I-II	232 (35.63)	232 (64.27)	0 (0.00)	<0.001 ^b^
III-IV	419 (64.37)	129 (35.73)	290 (100.00)	
Histological grade				
G1	30 (6.05)	19 (8.15)	11 (4.18)	<0.001 ^b^
G2	49 (9.88)	38 (16.31)	11 (4.18)	
G3	417 (84.07)	176 (75.54)	241 (91.63)	
Pathologica subtype				
Serous	225 (34.40)	187 (51.52)	38 (13.06)	<0.001 ^b^
Non-serous	429 (65.60)	176 (48.48)	253 (86.94)	
NACT				
No	528 (80.73)	318 (87.60)	210 (72.16)	<0.001 ^b^
Yes	126 (19.27)	45 (12.40)	81 (27.84)	

^a^Mann-Whitney test; ^b^Chi-square test;M: Median, Q_1_: 1st Quartile, Q_3_: 3st Quartile;.

^1^The cut-off value for CA125 is 264.5 U/mL;

^2^For postmenopausal patients, the cut-off value for HE4 is 148.5 U/mL; for premenopausal patients, the cut-off value is 122.2 U/mL;

^3^The cut-off value for ascites is 90 mL;

LNM, lymph node metastasis; FIGO, International Federation of Gynecology and Obstetrics; NACT, Neoadjuvant Chemotherapy.

**Table 2:** Univariate and multivariate logistic regression analysis.

Variables	Univariate analysis		Multivariate analysis	
	*P*	OR (95%CI)	*P*	OR (95%CI)
Stage (FIGO)				
I-II		1.00 (Reference)		1.00 (Reference)
III-IV	<0.001	519.75 (72.11 ~ 3746.07)	<0.001	519.75 (72.11 ~ 3746.07)
Tumor lateralization				
Unilateral		1.00 (Reference)		1.00 (Reference)
Bilateral	<0.001	3.83 (2.77 ~ 5.31)	0.015	1.83 (1.13~2.98)
NACT				
No		1.00 (Reference)		1.00 (Reference)
Yes	<0.001	2.73 (1.82 ~ 4.08)	0.049	0.60 (0.36 ~ 0.99)

OR, Odds Ratio; CI, Confidence Interval; FIGO, International Federation of Gynecology and Obstetrics; NACT, Neoadjuvant Chemotherapy.

### 4.2 Regional mapping of lymph node metastasis

After exclusion of patients who received neoadjuvant chemotherapy, a total of 210 lymph node–positive patients were included for regional mapping analysis. In total, 18,609 lymph nodes were resected, of which 2,015 were metastatic (LNM-positive); this yielded an overall lymph node positivity rate of 10.83%.

**Figure 2:**
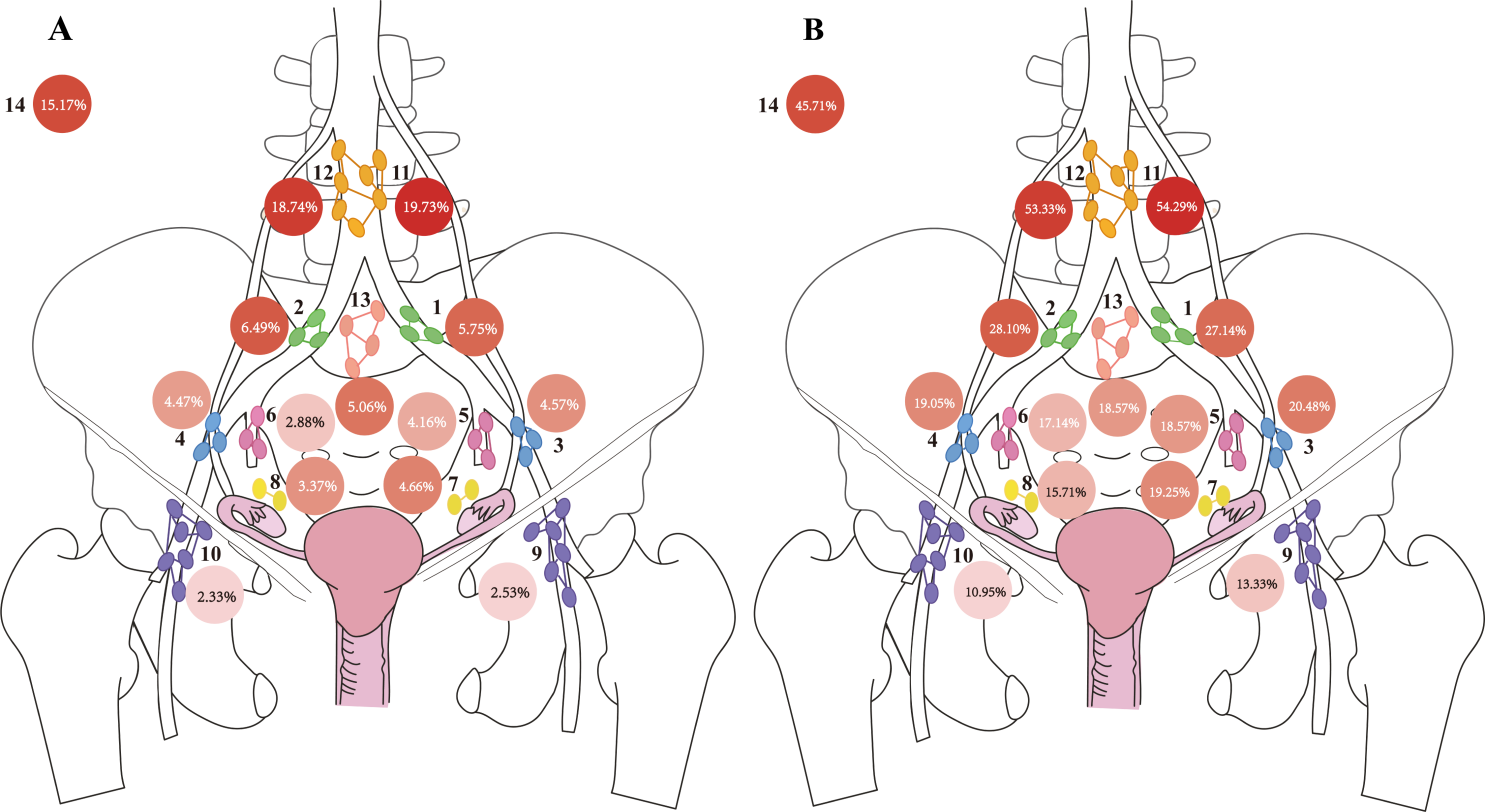
Regional distribution of lymph node (LN) positivity. Each circle represents a specific anatomical region. The number inside each circle indicates the proportion of LN positivity in that region, and the shading intensity of the circle corresponds to the magnitude of this proportion-darker shades indicate higher values. **(A)** distribution of 2,015 positive lymph nodes. The proportion is calculated as follows: (*number of positive LNs in the region)/(total number of positive LNs)*. **(B)** distribution of 210 patients with LNM. The proportion is calculated as follows: (*number of patients with positive LNs in the region)/(total number of patients with LNM)*.

All metastatic lymph nodes were classified into 14 anatomical regions according to established pelvic and para-aortic nodal anatomy (**Figure 1**). **Table 3** summarizes the frequency of lymph node dissection across these zones. For each zone, two key metrics were calculated: (1) the proportion of positive lymph nodes in that region relative to the total number of positive nodes, and (2) the proportion of patients with metastases in each anatomical zone. These distributions are presented in **Figure 2**.

**Table 3:** Number of removed lymph nodes.

Zone	Total number of lymph nodes removed (%) ^*^	Number of patients with lymph node removed (%) ^#^	Number of lymph nodes removed in each patient Median (minimum, maximum)
1	1077 (5.80)	383 (72.54)	1 (0, 19)
2	1131 (6.09)	380 (71.97)	2 (0, 14)
3	1083 (5.83)	372 (70.45)	1 (0, 17)
4	1069 (5.76)	362 (68.56)	1 (0, 14)
5	1044 (5.62)	361 (68.37)	1 (0, 17)
6	1000 (5.38)	350 (66.29)	1 (0, 13)
7	1481 (7.97)	355 (67.23)	2 (0, 21)
8	1641 (8.84)	349 (66.10)	2 (0, 25)
9	922 (4.96)	315 (59.66)	1 (0, 22)
10	946 (5.09)	312 (59.09)	1 (0, 13)
11	2821 (15.19)	410 (77.65)	4 (0, 46)
12	2427 (13.07)	376 (71.21)	3 (0, 37)
13	988 (5.32)	248 (46.97)	0 (0, 15)
14	979 (5.27)	225 (42.61)	0 (0, 32)

*Based on a total of 18609 lymph nodes removed.

#Based on all 528 patients.

As shown in **Figure 2A**, the left para-aortic lymph node region exhibited the highest proportion of positive nodes (19.73%), whereas the right superficial inguinal region had the lowest (2.33%). To more accurately reflect the metastatic propensity of each region, we calculated the ratio of patients with positive nodes in a given region to the total number of patients with any LNM. This metric served as an adjusted indicator of regional metastatic likelihood. **Figure 2B** shows that the left para-aortic region again demonstrated the highest ratio (54.29%), while the right superficial inguinal region remained the lowest (10.95%). Overall, para-aortic lymph nodes (Zones 11 and 12) emerged as the most common sites of metastasis, followed by bilateral pelvic nodal regions. In contrast, involvement of distal pelvic and superficial inguinal regions was relatively infrequent. The marked heterogeneity in regional involvement suggested non-random dissemination patterns along specific lymphatic routes.

**Figure 3:**
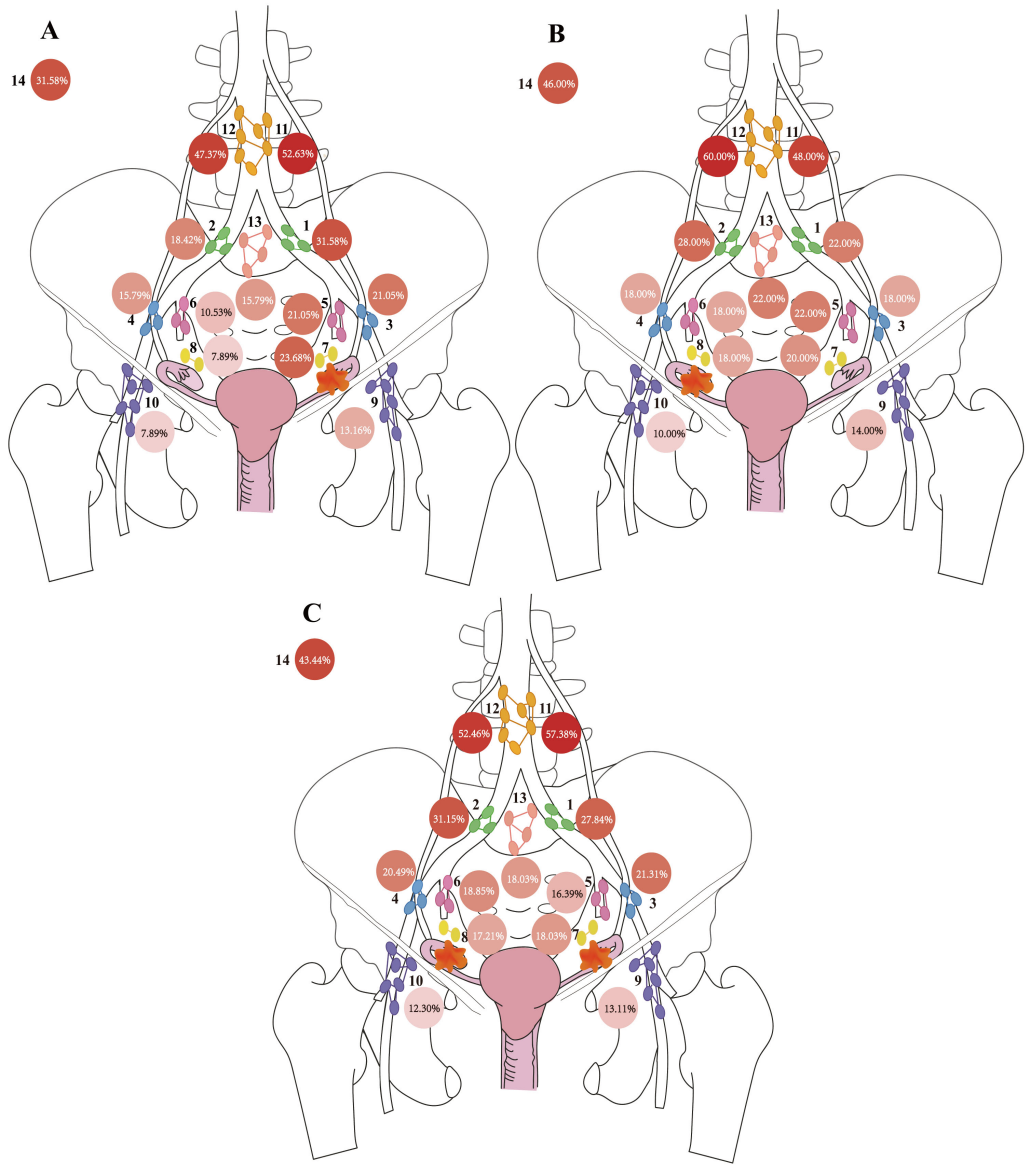
Regional distribution of lymph node (LN) metastasis ratios. Each circle represents a specific LN region. The number inside each circle indicates the proportion of patients with LNM in that region, calculated as follows: (*number of patients with positive LNs in the region)/(total number of patients with LNM)*. The shading intensity of each circle reflects the magnitude of the ratio, with darker shades indicating higher values. **(A)** distribution in patients with right-sided primary tumors. **(B)** distribution in patients with left-sided primary tumors. **(C)** distribution in patients with bilateral primary tumors.

Given that tumor lateralization was identified as an factor associated with LNM, the cohort was further stratified according to primary tumor lateralization into left-sided (n = 38), right-sided (n = 50), and bilateral tumors (n = 122). Across all subgroups, para-aortic regions consistently showed the highest metastatic involvement (**Figure 3**). Patients with unilateral tumors demonstrated a tendency toward higher metastasis rates in ipsilateral pelvic lymph nodes, whereas bilateral tumors were associated with a more symmetric distribution. These observations indicated that both anatomical proximity and laterality may influence regional metastatic spread.

### 4.3 Bayesian and co-occurrence probability calculations

**Figure 4:**
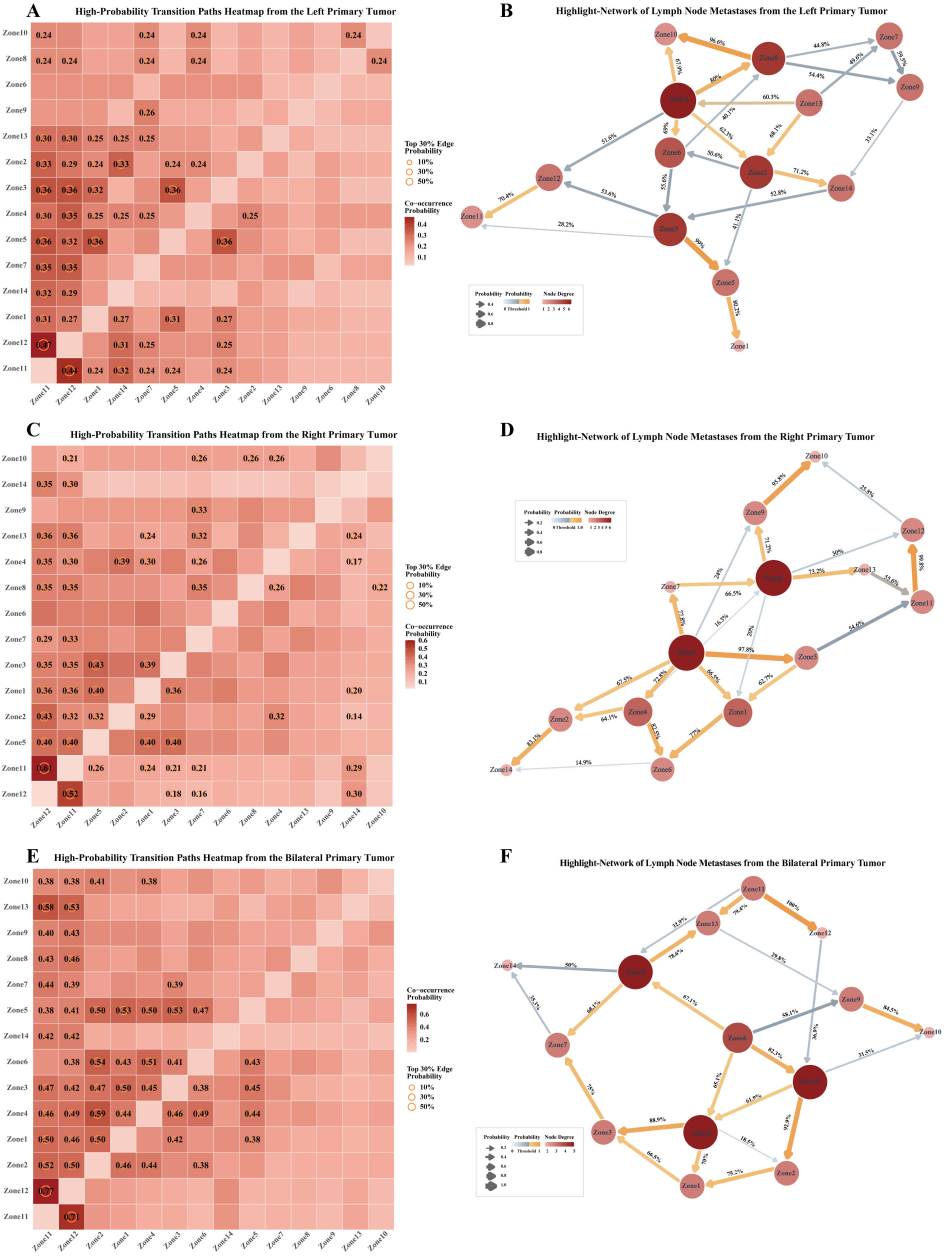
Co-occurrence probability heatmaps and Bayesian network diagrams of LNM across anatomical regions. **(A, C, E)** present heatmaps illustrating the top 30% highest co-occurrence probabilities of lymph node metastasis (LNM) between pairs of anatomical regions. Each square represents the probability of concurrent metastasis in two regions, with darker colors indicating higher probabilities. Circles mark the top 30% co-occurrence probabilities, and larger circles correspond to higher values. **(B, D, F)** display the corresponding Bayesian networks. Each node represents an anatomical region of lymph nodes (LN), and directed edges denote conditional dependencies (i.e., potential causal relationships) between regions. The color intensity and size of each node reflect its connectivity: darker and larger nodes indicate greater influence within the network. The thickness and color depth of edges are proportional to the strength of the Bayesian probability. Edges with probabilities > 0.6 are displayed with a gradient color to highlight likely metastatic pathways. **(A, B)** Data from patients with left-sided primary tumors; **(C, D)** Data from patients with right-sided primary tumors; **(E, F)** Data from patients with bilateral tumors.

To further explore potential lymphatic metastasis patterns, inter-regional relationships among lymph node zones were assessed using both co-occurrence probabilities and Bayesian conditional probabilities. Heatmaps of co-occurrence probabilities reflect the likelihood that any two regions are simultaneously involved in metastasis, while Bayesian network diagrams illustrate the inferred probabilistic associations between regions, with edge weights corresponding to association strength (**Figure 4**). Across all heatmaps stratified by tumor lateralization (**Figures 4A, C, E**), the left and right para-aortic zones (Zones 11 and 12) formed high-frequency co-occurring pairs, whereas Zones 9, 10, and 14 exhibited low co-occurrence frequencies regardless of tumor laterality. The Bayesian network diagrams (**Figures 4B, D, F**) further revealed that Zones 1, 10, 12, 13, and 14 are terminal nodes, while Zones 3, 4, 5, 6, and 8 function as central hubs with extensive connections to multiple other regions–indicating their potential role in lymphatic spread. In addition, patients with left-sided primary tumors exhibited a clear tendency for ipsilateral metastasis, following the specific pathway: Zone 3 → Zone 5→ Zone 1. In contrast, patients with right-sided primary tumors did not demonstrate a clear lateralization trend, and their metastatic patterns were similar to those of patients with bilateral primary tumors, which were characterized by widespread cross-metastasis within the pelvic lymph nodes (Zones 1-8).

### 4.4 Crossing-pathways analysis and visualization

**Figure 5:**
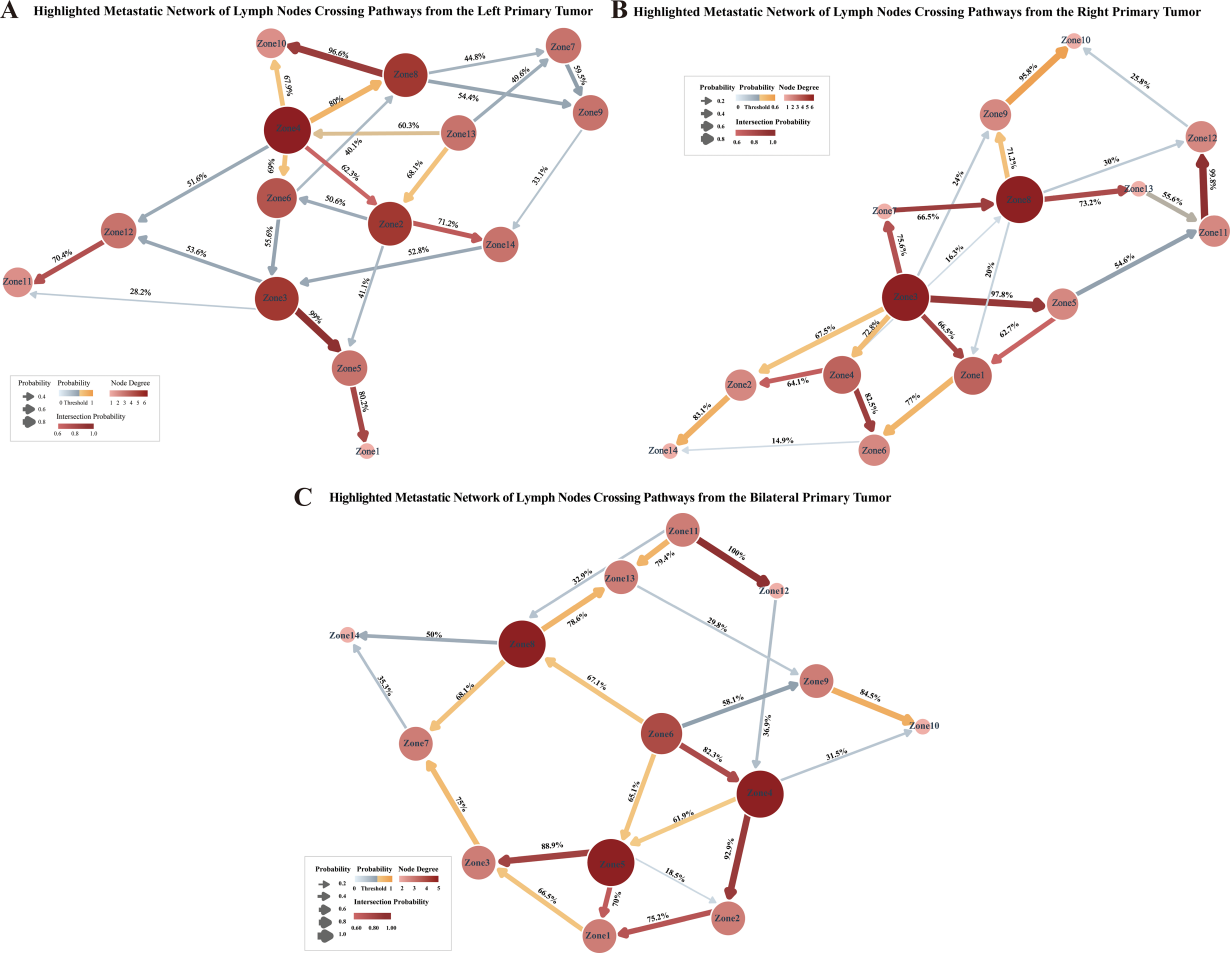
Highlighted Bayesian network diagrams of potential cross-regional metastatic pathways. Each node represents a lymph node (LN) anatomical region, and directed edges indicate conditional dependencies based on bootstrap-derived Bayesian posterior probabilities. The direction of the arrows denotes the likely causal direction of metastasis. Node color and size reflect network connectivity: nodes with more outgoing or incoming connections appear larger and darker. The color and thickness of edges are proportional to the posterior probability, with darker and thicker lines indicating higher probabilities. Edges with posterior probabilities > 0.6 are highlighted with a yellow gradient, signifying likely metastatic pathways. Edges that meet both criteria—posterior probability > 0.6 and co-occurrence probability in the top 30%—are emphasized with a red gradient, representing potential cross-regional key transitions. **(A)** patients with left-sided primary tumors​; **(B)** patients with right-sided primary tumors​; **(C)** patients with bilateral primary tumors.

To identify anatomically plausible metastatic pathways, two criteria were applied: 1) bootstrap-derived Bayesian edge strength ≥60%, and 2) co-occurrence probability within the top 30%. Connections meeting both criteria were defined as high-probability metastasis routes and highlighted on the Bayesian network diagram (**Figure 5**). Notably, to maintain biological plausibility, we deliberately excluded connections between anatomically distant regions and did not label such connections.

In patients with left-sided primary tumors (**Figure 5A**), high-probability pathways included: i) Zone 8→ Zone 10, ii) Zone 4→ 2→ 14, Zone 3→ 5→ 1, and iii) Zone 12→ 11. The pathways predominantly originate from Zones 3, 4, and 8, and terminate in Zones 1, 10, 11, and 14. Pelvic lymph node metastasis exhibited a certain lateralization tendency, with preferential spread to ipsilateral nodes. However, certain pathways suggested possible cross-lateral dissemination–particularly involving the para-aortic region–where metastases may first spread to contralateral lymph nodes and subsequently return to nodes ipsilateral to the primary tumor. This pattern may reflect complex retrograde or translineal lymphatic drainage. In patients with right-sided primary tumors (**Figure 5B**), several metastatic pathways were observed: i) Zone 3→ 7→ 8→ 13, ii) Zone 4→ 2, Zone 4→ 6, Zone 3→ 5→ 1, and iii) Zone 11→ 12. These pathways predominantly originated from Zones 3 and 4, terminating in Zones 1, 2, and 12. A certain lateralization trend was observed in the metastasis of pelvic lymph nodes in patients with right-sided primary tumors. In patients with bilateral primary tumors (**Figure 5C**), the following routes were identified: i) Zone 6 →4 →2 →1; ii) Zone 5 →1; iii) Zone 5 →3; and iv) Zone 11 →12. These pathways mainly originated from Zones 5 and 6, with endpoints at Zones 1, 10, 12, and 14. The metastatic paths of patients with bilateral tumors involved multiple regions of the left and right pelvic cavities, exhibiting a multicentric and confluent pattern of lymphatic spread. This contrasts with the relatively preserved unilateral metastatic pattern observed in patients with unilateral tumors, reflecting more extensive and widespread lymphatic drainage in bilateral tumors.

Based on the above findings, several key conclusions can be drawn:

1. Most pelvic lymph node metastases follow the principle of “ipsilateral priority, spreading along the chain of anatomical structures”. In contrast, bilateral primary tumors exhibit the characteristics of “fusion-type spread, multiple pathways, and multiple endpoints”.
2. Three distinct metastatic pathways were inferred:

- Iliac convergence pathway: Internal (Zones 5–6) and external iliac (Zones 3–4) nodes converge at common iliac nodes (Zones 1–2), serving as terminal stations.
- Para-aortic primary pathway: Para-aortic nodes (Zones 11–12) often show isolated involvement, with potential cross-lateral spread, in which contralateral para-aortic nodes are involved first, after which metastasis may potentially spread back to the side ipsilateral to the primary tumor.
- Obturator hub pathway: Obturator nodes (Zones 7–8) act as central hubs, potentially facilitating onward spread to inguinal (Zones 9–10) and presacral nodes (Zone 13).

These probabilistic maps provide a structured overview of the likely lymphatic dissemination patterns in EOC, highlighting central hubs, terminal nodes, and lateralization tendencies.

### 4.5 Bayesian network model incorporating clinical factors

**Figure 6:**
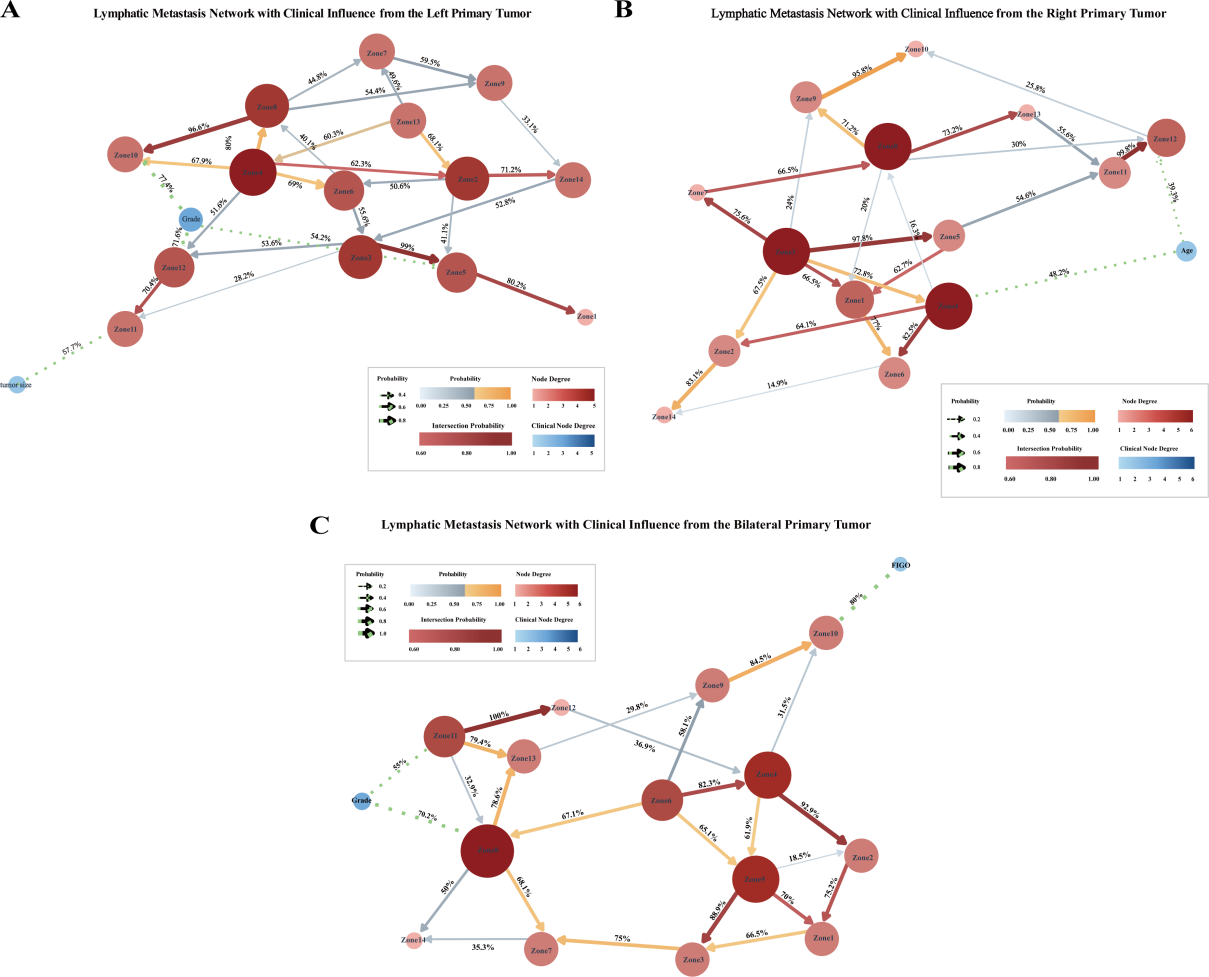
Highlighted Bayesian network diagrams with clinical factors. Each node represents either a lymph node (LN) anatomical region or a clinical variable. Directed edges indicate bootstrap-derived Bayesian posterior probabilities between nodes, with arrows denoting the inferred direction of influence. Node size and color intensity reflect the level of connectivity: nodes with more interactions appear larger and darker. The thickness and color of edges correspond to the magnitude of the posterior probability, with thicker and darker edges indicating stronger associations. Associations involving clinical factors are represented by green dashed lines.​ **(A)** patients with left-sided primary tumors; **(B)** patients with right-sided primary tumors​; **(C)** patients with bilateral primary tumors.

To further evaluate the potential influence of clinical factors on the likelihood of different metastatic pathways, eight key variables were incorporated into a Bayesian network model: age, menstrual status, serum concentrations of CA125 and HE4, FIGO stage, histological grade, tumor size, and pathological subtype. Variables that demonstrated significant associations with metastatic routes were retained and visualized in the network diagram (**Figure 6**). For patients with left-sided primary tumors, tumor size and histological were found to influence metastasis to para-aortic (Zones 11–12) and inguinal lymph nodes (Zone 10). Conditional probability analysis (**Supplementary Table 1**) indicated that the probability of metastasis to Zone 11 increased with tumor size, even after accounting for Zone 12 status. Zone 12 involvement correlated positively with higher histological grade, while Zone 10 metastasis was influenced jointly by Zone 8 involvement and tumor grade, suggesting that aggressive tumor biology may promote distal dissemination. The transition probability from Zone 3 to Zone 5 was strongly grade-dependent, with Zone 5 metastasis observed in 87.5% of Grade 3 cases with Zone 3 involvement, indicating a stepwise pattern along pelvic lymphatic chains.

For patients with right-sided primary tumors, age emerged as a key determinant of para-aortic involvement. Patients under 50 years exhibited a 62.5% probability of Zone 12 metastasis in the absence of Zone 11 involvement, whereas older patients’ risk increased markedly from 16% to 88.2% when Zone 11 was affected. Age also influenced Zone 2 involvement, with younger patients showing higher likelihood independent of Zone 4 status, suggesting an age-related progression-dependent pattern.

Among patients with bilateral primary tumors, both FIGO stage and histological grade influenced the probability of metastasis to para-aortic and inguinal regions. Zone 11 involvement increased with tumor grade, reaching 61.9% in Grade 3 tumors. Zone 8 involvement was highest in Grade 2 tumors but lower in Grade 3, and advanced FIGO stage (Stage IV vs. III) was associated with higher probability of Zone 10 metastasis (27.6% vs. 7.5%).

In conclusion, clinical factors, particularly tumor size, grade, and patient age, play a critical role in shaping the lymphatic metastatic patterns of patients with EOC. Tumor size is directly associated with the risk of para-aortic lymph node metastasis, with larger tumors demonstrating a significantly higher probability of direct metastasis–sometimes bypassing intermediate nodal stations. Higher-grade tumors exhibited increased involvement of distant lymph node, while younger patients tended toward sequential or ascending spread. Specific sequential patterns identified (Zone 12 → 11, Zone 8 → 10, Zone 3 → 5) underscore how tumor biology and patient characteristics jointly shape metastatic trajectories. Collectively, these findings provide a framework for individualized risk assessment and could inform targeted lymphadenectomy strategies in EOC.

## 5 Discussion

Lymph node metastasis (LNM) is a core factor influencing staging determination and prognostic evaluation of epithelial ovarian cancer (EOC). Although systematic lymphadenectomy is a commonly used clinical procedure, its survival benefit remains controversial due to an increased risk of surgical complications (9, 10). Prior studies have mainly focused on local nodal involvement, lacking systematic analyses of metastatic pathways and intuitive visual models of lymphatic spread. This has led to variability in clinical decision-making regarding the extent of lymphadenectomy and adjuvant treatment. Against this background, a comprehensive characterization of lymphatic dissemination patterns and their clinical correlates remains lacking in EOC. In the era of effective systemic therapies, the role of lymph node assessment has shifted from a purely surgical consideration to a component of disease characterization, which may contribute to risk stratification and biological understanding. Therefore, this study aimed to provide a probabilistic and anatomically informed description of lymph node involvement patterns.

The results of this study revealed that LNM in EOC exhibits a “global” feature, involving all anatomical regions, with the highest metastasis rate in para-aortic lymph nodes (Zone 11,54.29%). This is consistent with the findings of Kleppe et al. (35), who observed “80% para-aortic lymph node involvement in early-stage EOC patients,” and Euscher et al. (36), who reported “85.7% para-aortic metastasis in patients with positive sentinel lymph nodes.” More importantly, to our knowledge, this study is the first to construct a visual model of EOC lymphatic metastasis using Bayesian network modeling in a clinical patient cohort. Using a Bayesian network framework, we identified recurrent patterns of nodal co-occurrence and conditional dependency across anatomical regions. These patterns should be interpreted as probabilistic associations rather than definitive metastatic routes. For example, we observed that pelvic nodal regions (e.g., internal/external iliac nodes) were frequently associated with common iliac involvement, while para-aortic regions demonstrated complex bilateral associations. This finding is anatomically consistent with the three ovarian lymphatic drainage pathways confirmed by Pal et al. (37) in animal models. Unlike previous animal experiments or cadaveric studies (35, 37), this study further clarified the association strength between lymph node subregions and intermediate transition nodes (e.g., common iliac lymph nodes, obturator lymph nodes) in metastatic pathways, thereby helping bridge the gap between anatomical observations and clinically observed metastatic patterns.

An interesting observation in this study was the presence of cross-regional involvement between para-aortic lymph nodes, including contralateral and bilateral patterns. This phenomenon is supported by the case report of Nomura et al. (38), which described left-sided ovarian tumors with isolated right-sided para-aortic metastasis, and the cohort study of Pereira et al. (39), in which 7% of patients with only contralateral para-aortic involvement and 40% with bilateral involvement. These observations suggest that para-aortic lymphatic dissemination in EOC does not strictly adhere to an ipsilateral-priority model. Instead, cross-metastasis and bilateral involvement appear to represent non-negligible patterns, underscoring the complexity of ovarian lymphatic spread. From an anatomical perspective, this behavior may be explained by the bidirectional lymphatic drainage from the ovary to the para-aortic region via the suspensory ligament. However, given the retrospective design and the modeling approach used, these findings should be interpreted as hypothesis-generating observations.

In terms of risk factor analysis, this study confirmed associations with several clinicopathological factors previously reported, such as tumor laterality, pathological subtype, International Federation of Gynecology and Obstetrics (FIGO) stage, positive peritoneal cytology, histological grade, and serum CA125 level. Importantly, our analysis further suggests that different clinical factors may be associated with distinct aspects of nodal disease, including not only the presence of metastasis but also its anatomical distribution. For example, in the full cohort, smaller tumor size was observed in patients with lymph node metastasis, a finding that has also been reported in previous studies (40). In contrast, among patients with pathologically confirmed nodal metastasis, larger tumor size was associated with an increased probability of para-aortic involvement in the Bayesian analysis. This apparent discrepancy likely reflects the distinction between factors associated with the occurrence of nodal metastasis and those influencing the anatomical distribution or extent of metastatic spread. Consistent with previous reports (40), larger tumor burden and higher tumor grade may be associated with more extensive or anatomically advanced nodal involvement. In addition, younger patients in our cohort showed a tendency toward more widespread nodal involvement, although this observation should be interpreted with caution and warrants further validation.

Despite the widespread use of systematic lymphadenectomy for surgical staging in epithelial ovarian cancer, large-scale clinical studies and randomized trials have reported inconsistent survival benefits, while highlighting increased surgical morbidity (41).These findings underscore the limitations of uniform nodal dissection strategies and emphasize the need for a more refined understanding of lymphatic dissemination patterns to guide surgical decision-making. From the perspective of clinical translation, the results of this study may help inform surgical planning and adjuvant treatment considerations in EOC. The findings of this study are not intended to directly guide surgical decision-making or to replace existing clinical guidelines. Rather, they provide a probabilistic framework that may help generate hypotheses for future studies investigating risk-adapted nodal assessment strategies. For instance, the observed predominance of para-aortic involvement and bilateral associations may warrant further investigation in prospective settings to determine whether selective nodal evaluation strategies can be optimized. The visual network of metastatic pathways may facilitate hypothesis-driven, targeted lymphadenectomy strategies, optimizing the balance between complete resection and minimizing surgical morbidity. In radiotherapy, knowledge of high-risk metastatic routes (e.g., para-aortic cross-transfer, obturator-to-inguinal) may inform precise delineation of target volumes, reducing unnecessary exposure to normal tissue. These findings are not intended to replace existing surgical guidelines, but rather to provide complementary, pathway-based evidence to inform individualized nodal assessment.

Despite these valuable insights, our study has several limitations. First, this was a single-center retrospective study, and the findings may be influenced by institutional surgical practices and patient selection, which may limit generalizability. Second, the Bayesian network model is dependent on the structure and quality of the input data, and the inferred relationships reflect conditional dependencies rather than causal mechanisms. Third, although efforts were made to reduce bias by excluding patients who received neoadjuvant chemotherapy in the pathway analysis, residual confounding cannot be excluded. Future studies, particularly multi-center prospective investigations integrating imaging and molecular data, are warranted to validate and extend these findings.

## 6 Conclusion

This study represents the first attempt to construct a visual, probabilistic model of EOC lymphatic metastasis in a clinical cohort using Bayesian network modeling, clarifying three parallel metastatic pathways and the “cross-metastasis” pattern, identifying preoperative neoadjuvant chemotherapy as an protective factor, and revealing the guiding role of clinical characteristics in metastatic distribution. These findings contribute to the conceptual understanding of EOC lymphatic dissemination and provide complementary, pathway-based evidence that may inform precision-oriented nodal assessment and individualized adjuvant treatment, thereby supporting a gradual shift toward more anatomy- and pattern-informed clinical decision-making in EOC.

## Data Availability

The raw data supporting the conclusions of this article will be made available by the authors, without undue reservation.

## Supplementary material

Supplementary Figure 1 Flowchart of the patient selection process. A total of 654 patients with epithelial ovarian cancer (EOC) were initially included. According to the presence or absence of lymph node metastasis (LNM), these patients were divided into two groups: the LNM-positive (LN+) group and the LNM-negative (LN–) group, for subsequent analysis. Among the 654 patients, 210 were further selected for lymph node mapping analysis based on inclusion criteria derived from logistic regression.
