# Supplementary figures and images for: Probabilistic mapping of lymph node metastasis in epithelial ovarian cancer: a retrospective cohort study using Bayesian network analysis
Source: Front Oncol. 2026 May 18;16:1817368. doi: 10.3389/fonc.2026.1817368 (PMC13223166; doi:10.3389/fonc.2026.1817368)

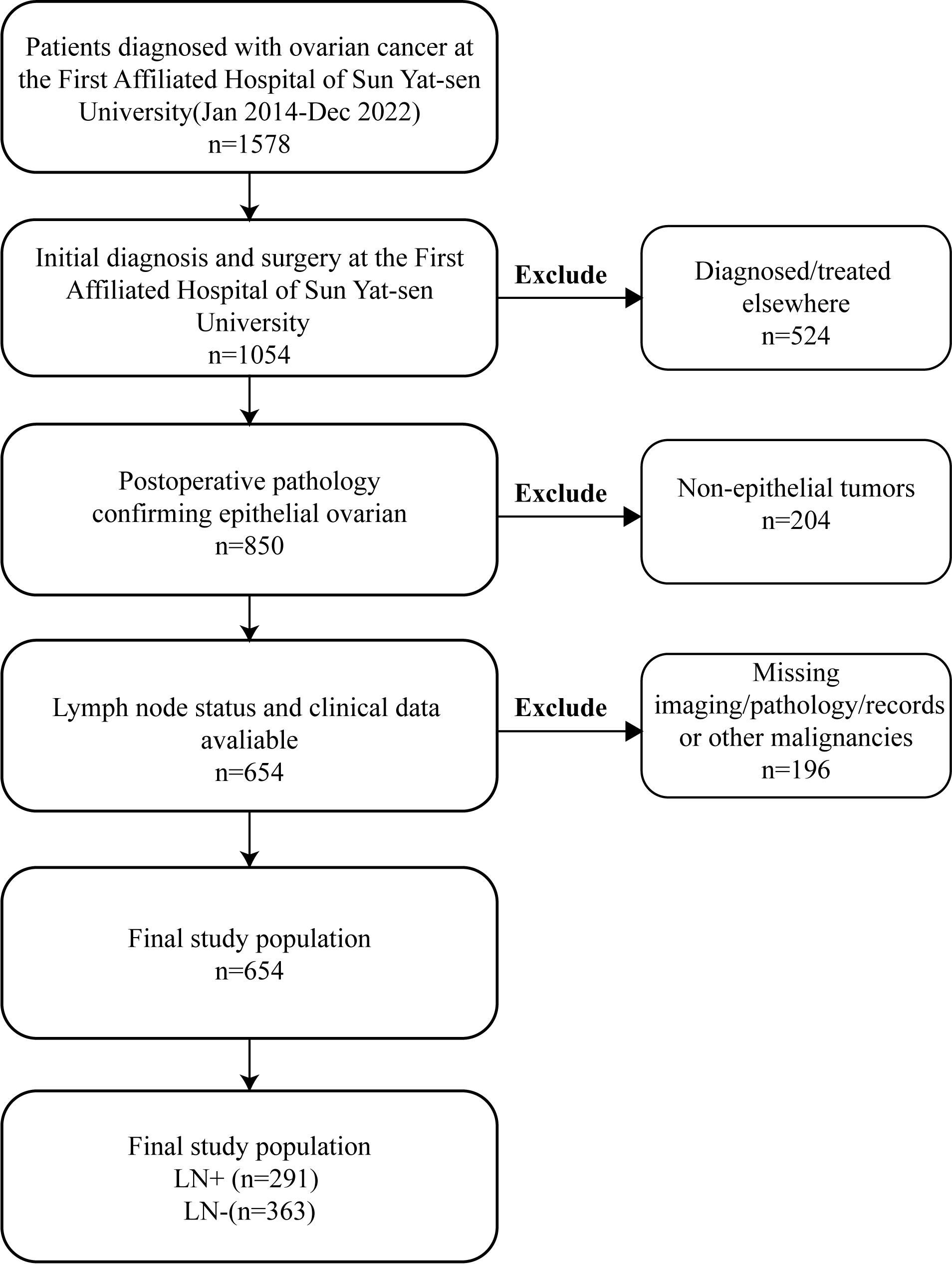

Supplement: Supplementary Figure 1 — Flowchart of the patient selection process. A total of 654 patients with epithelial ovarian cancer (EOC) were initially included. According to the presence or absence of lymph node metastasis (LNM), these patients were divided into two groups: the LNM-positive (LN+) group and the LNM-negative (LN–) group, for subsequent analysis. Among the 654 patients, 210 were further selected for lymph node mapping analysis based on inclusion criteria derived from logistic regression. [file Image1.tif]
