# Supplementary material for: Probabilistic mapping of lymph node metastasis in epithelial ovarian cancer: a retrospective cohort study using Bayesian network analysis
Source: Front Oncol. 2026 May 18;16:1817368. doi: 10.3389/fonc.2026.1817368 (PMC13223166; doi:10.3389/fonc.2026.1817368)
Supplement: Supplementary file 2 [file Table1.docx]

**Supplement Table 1: Conditional probabilities of lymph node metastasis under clinical factors.**

| Lymph Node | Tumor lateralization | Clinical Factor(s)^1^ | Condition Level(s)^2,3^ | P(Metastasis)^4^ | Trend^5^ |
| --- | --- | --- | --- | --- | --- |
| Zone11 | Left | Tumor size & Zone12 status | Size = 1, Z12 = Absent | 0.00% | ↑ with size |
|  |  |  | Size = 2, Z12 = Absent | 11.10% |  |
|  |  |  | Size = 3, Z12 = Absent | 28.60% |  |
|  |  |  | Size = 4,Z12 = Absent | 100.00% |  |
|  |  |  | Size = 1, Z12 = Present | 100.00% | Present if Z12 is involved |
|  |  |  | Size = 2, Z12 =Present | 60.00% |  |
|  |  |  | Size = 3, Z12 = Present | 100.00% |  |
|  |  |  | Size = 4, Z12 = Present | 100.00% |  |
| Zone12 | Left | Grade | Grade 1 | 50.00% |  |
|  |  |  | Grade 2 | 0.00% |  |
|  |  |  | Grade 3 | 53.10% | Highest in Grade 3 |
| Zone10 | Left | Grade & Zone8 status | Grade 3, Z8 = Absent | 0.00% | Present in Grade 3 only if Z8 is involved |
|  |  |  | Grade 3, Z8 = Present | 100.00% |  |
| Zone5 | Left | Grade & Zone3 status | Grade 2 | 25.00% |  |
|  |  |  | Grade 3, Z3 = Absent | 0.00% | ↑ When Z3 is involved in Grade 3 |
|  |  |  | Grade 3, Z3 = Present | 87.50% |  |
| Zone12 | Right | Age group & Zone11 status | Young, Z11 = Absent | 62.50% | Young > Elderly |
|  |  |  | Elderly, Z11 = Absent | 16.70% |  |
|  |  |  | Young, Z11 = Present | 100.00% |  |
|  |  |  | Elderly, Z11 = Present | 88.20% |  |
| Zone2 | Right | Age group & Zone4 status | Young, Z4= Absent | 8.30% | Young > Elderly |
|  |  |  | Elderly, Z4 = Absent | 0.00% |  |
|  |  |  | Young, Z4 = Present | 100.00% |  |
|  |  |  | Elderly, Z4= Present | 45.50% |  |
| Zone11 | Bilateral | Grade | Grade 1 | 42.90% | ↑ With Grade |
|  |  |  | Grade 2 | 20.00% |  |
|  |  |  | Grade 3 | 61.90% |  |
| Zone8 | Bilateral | Grade | Grade 1 | 28.60% | Peak at Grade 2 |
|  |  |  | Grade 2 | 40.00% |  |
|  |  |  | Grade 3 | 14.30% |  |
| Zone10 | Bilateral | FIGO stage | Stage III | 7.50% | ↑ With stage |
|  |  |  | Stage IV | 27.60% |  |

1: Age group: Young < 50 years, Elderly ≥50 years.

Grade: 1= well-differentiated, 2= moderately differentiated, 3= poorly/undifferentiated.

Tumor size: Size = 1: <50 mm; Size = 2: 50–100 mm; Size = 3: 100–150 mm; Size = 4: >150mm.

FIGO: International Federation of Gynecology and Obstetrics

2: “Present” and “Absent” indicate whether the referenced lymph node region was involved in metastasis. Conditional probabilities were calculated as the observed proportion of metastases in each zone given the specified clinical scenario.

3: Zx = Zonex (e.g., Z12 = Zone12), "Z#" = Present/Absent indicates whether lymph node metastasis is present in the corresponding zone.
4: Probabilities represent P(metastasis) under specified conditions.

5: "↑" indicates an increasing trend in metastasis probability with higher levels of the listed clinical factor. "Peak" refers to a local maximum or minimum in metastasis probability at a specific level.
